# Continuation Rates of the Etonogestrel Implant and Factors Associated With Early Discontinuation

**DOI:** 10.7759/cureus.36117

**Published:** 2023-03-14

**Authors:** Genesis Hines, Carrie Wang, Treasure Walker, Amyeo Jereen, Joanne N Quinones, Andrea Waxman

**Affiliations:** 1 Obstetrics and Gynecology, Lehigh Valley Health Network, Allentown, USA; 2 Obstetrics and Gynecology/University of South Florida Morsani College of Medicine, Lehigh Valley Health Network, Allentown, USA

**Keywords:** long-acting contraception, unplanned pregnancy, side effects, abnormal uterine bleeding, contraception, etonogestrel implant

## Abstract

Background: The etonogestrel implant is generally considered an effective, three-year, long-acting reversible contraceptive device. Previous research, such as the landmark CHOICE study, has reported a one-year continuation rate of 72% to 84%, however, in a real-world setting these rates may be significantly lower.

Objective: To study etonogestrel implant continuation rates and factors associated with early discontinuation in a specific clinical setting.

Study design: Single-center, retrospective cohort study of patients who received the etonogestrel implant between January 1, 2015, and December 31, 2017, at several practices at an academic community hospital network. Records were reviewed up to three years after implant insertion to determine continuation rates (one to three years), early discontinuation rates (≤12 months), and reasons for early discontinuation. A sample size calculation was performed to guide a subanalysis of side effects.

Results: A total of 774 patients underwent etonogestrel insertion during the study period. The one-year continuation rate was lower than that of the CHOICE study (62% vs. 83%, P <0.001). A subanalysis (n=216) revealed that a majority (82%, n=177) of patients reported side effects. Side effects were more common in patients with early discontinuation compared with patients who continued use longer than one year (93% vs. 71%, P <0.001). The most common side effect, abnormal uterine bleeding, was not significantly associated with early discontinuation. A significant association (P=0.02) was found between early discontinuation and neurologic/psychiatric complaints.

Conclusions: The one-year continuation rate of the etonogestrel implant in our population is significantly lower than the value reported by CHOICE. Implant side effects are common and significantly affect rates of discontinuation. Our data suggest there is an opportunity for education and counseling for individuals opting for this method of long-acting contraception.

## Introduction

Long-acting reversible contraception (LARC) is the gold standard and first-line contraception choice for patients seeking to avoid pregnancy in the next 12 months [1-3]. Entities such as the World Health Organization (WHO) and the American College of Obstetricians and Gynecologists (ACOG) assert that all eligible patients should be counseled on LARCs, as these methods remain the most effective method of preventing pregnancy [1]. LARCs include intrauterine devices (IUDs) and contraceptive implants.

The etonogestrel implant, a subdermally implanted hormonal rod, is a highly effective form of birth control that can prevent pregnancy for up to three years. With an annual pregnancy rate of just 0.05%, this implant is considered superior to non-LARC methods such as short-acting hormonal pills, patches, and barrier methods [2]. Both LARC insertion and removal are a matter of patient autonomy, thus highlighting the importance of understanding side effects (such as abnormal bleeding) that may impact the decision to proceed with this contraceptive choice. The most common side effect is irregular menstrual bleeding; according to the NEXPLANON website, this side effect caused one in 10 patients in preliminary studies to discontinue the device [4]. A 2021 multi-country meta-analysis by Moray et al. found that the implant has a lower one-year continuation rate than its IUD counterparts (77.5% vs. 84.8% for the levonorgestrel IUD and 83% for the copper IUD) [5]. Several European studies have suggested that overall continuation rates vary between 74% and 84% in the first 12 months [6,7]. The CHOICE project, one of the largest US-based studies of etonogestrel implant continuation N=9,256, found a one-year continuation rate of 83% among patients ages 14 to 45 who were given LARC counseling and free access to contraception [8]. Other US studies have reported similar rates of continuation [9,10].

Given the importance of effective contraception and appropriate counseling for patients wishing to avoid unintended pregnancy, our study sought to determine how the one-year implant continuation rates in a population at an academic community hospital network compare with national and international findings as well as determine what factors are associated with early discontinuation.

## Materials and methods

We performed a single-center retrospective cohort study of individuals >18 years of age with documented etonogestrel implant insertions at the practices of the Department of Obstetrics and Gynecology at Lehigh Valley Health Network, Allentown, Pennsylvania, between January 1, 2015, and December 31, 2017. Patient demographics, medical history, insertion and removal documentation, side effects, treatment, and outcome data were extracted from the electronic medical records for analysis. Patient data were deidentified and stored in a secure password-protected spreadsheet. Follow-up data from outpatient visits were obtained for up to three years after placement of the implant. Patients without documented implant removal within 38 months were excluded from the study as lost to follow-up, as the implant is only approved for use by the Food and Drug Administration for a three-year period.

Our primary objective was to describe the continuation rates of the implant in our patient population. We defined early discontinuation as removal at less than or equal to one year after insertion. The secondary objective was to identify factors significantly associated with early discontinuation. The factors included in the analysis were an a priori list of commonly reported reasons patients gave for requesting early removal of the implant.

We analyzed a subset of the population to identify factors associated with early discontinuation. To determine the subset sample size, we hypothesized, based on our general clinical experience, that the one-year continuation rate of the etonogestrel implant in our population was at least 23% lower (a statistically and clinically significant reduction) than the CHOICE study outcome of 83%. The statistical power was set at 90%, and statistical significance was set at P <0.05. The sample size analysis indicated that 108 patients were needed in each of the two comparison groups - patients who discontinued use at ≤1 year and patients who continued use for >1 year. Individuals were randomly selected for a total sample of 216 patients.

Comparisons were made using Student’s t-tests for continuous variables, and chi-square analyses or Fisher exact tests for categorical variables. Significance was set at P<0.05. Stata/SE version 16.0 software (StataCorp LLC, College Station, TX) was used for statistical analysis. The study was approved by the health network’s institutional review board.

## Results

**Figure 1:**
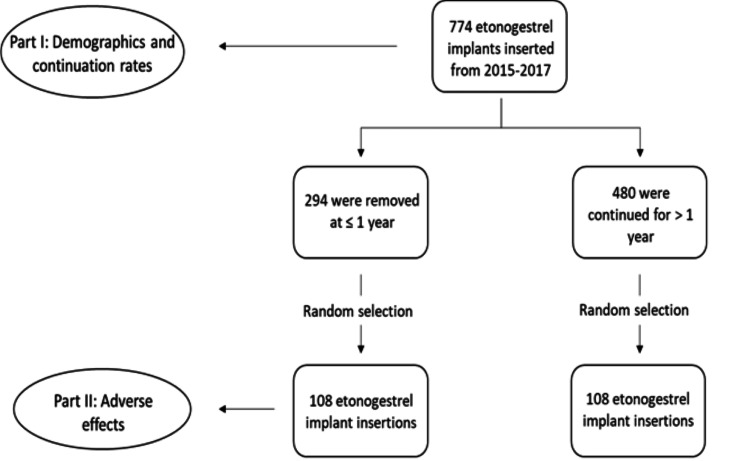
Population selection and study design

**Figure 2:**
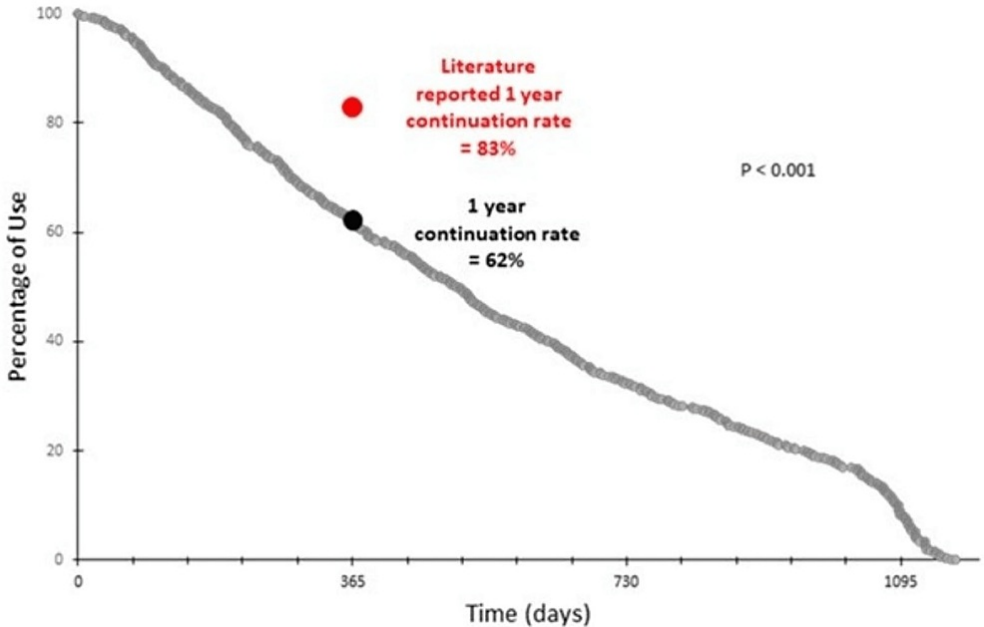
Etonogestrel implant continuation rates over time

During the study period, 774 patients at our institution underwent placement of an etonogestrel implant (Figure 1). The mean age of our population was 24.3 years (+ 5.8) and most patients identified as White (62.5%). Insurance coverage was almost evenly split between government insurance (49.0%) and private insurance (49.6%). Table 1 displays the demographics of our study population categorized by continuation category. Patients who discontinued the implant earlier were younger when compared to women who continued the implant beyond one year (23.7 years vs. 25.3 years, P <0.001). No other statistically significant differences were noted between the groups. The one-year etonogestrel implant continuation rate was 62%, which was significantly lower (P <0.001) than the CHOICE study’s reported value of 83% (Figure 2). The two- and three-year continuation rates were 32% and 8%, respectively.

**Table 1:** Baseline characteristics of the entire population by etonogestrel continuation rates Data were analyzed using Student’s t-tests, chi-square, or Fisher exact tests as indicated. ^a^Data are expressed as mean ± standard deviation.

Demographics	Continuation for ≤ 1 year (n=294)	Continuation >1 year (n=480)	P value
Age (years)^a^	25.3 ± 6.1	23.7 ± 5.5	<0.001
Race, n (%)			
Black	30 (10.2)	36 (7.5)	0.48
White	183 (62.2)	301 (62.7)	
Other	73 (24.8)	133 (27.7)	
Missing	8 (2.7)	10 (2.1)	
Hispanic Ethnicity, n (%)	118 (40.3)	225 (47.4)	0.06
Relationship Status, n (%)			
Single	213 (73.0)	372 (77.7)	0.14
Married	79 (27.1)	107 (22.3)	
Body mass index (kg/m^2^)^a^	28.4 ± 7.1	28.9 ± 7.1	0.46
Insurance Type, n (%)			
Government	139 (47.3)	240 (50.0)	0.73
Private	151 (51.4)	233 (48.5)	
Other	4 (1.4)	7 (1.5)	
Prior pregnancy, n (%)	198 (70.5)	313 (69.6)	0.8
Prior etonogestrel implant use, n (%)	4 (1.4)	10 (2.1)	0.58

In the subanalysis of 216 randomly selected patients (Table 2), 73% (n=158) of patients experienced adverse effects. Patient-reported adverse effects were significantly associated with early discontinuation (89.8% vs. 57.0%, P < .001). No other patient-reported factors were significantly associated with early discontinuation.

**Table 2:** Reported reasons for etonogestrel implant discontinuation by continuation rates Data were analyzed using chi-square, or Fisher exact tests as indicated.

Reported reason for discontinuation	Continuation for ≤1 year (n=108)	Continuation > 1 year (n=108)	P value
Desired pregnancy, n (%)	6 (5.6)	14 (13.1)	0.06
Adverse effects, n (%)	97 (89.8)	61 (57.0)	<0.001
Desire different therapy, n (%)	3 (2.8)	4 (3.7)	0.72
Other, n (%)	2 (1.9)	1 (0.09)	1.0

Looking more closely at the adverse effects data (Table 3), 81.9% (n=177) of patients in the subset analysis reported experiencing adverse effects. The most common side effect was abnormal uterine bleeding (AUB), reported by 53% of patients (n=115). AUB, however, was not significantly associated with early discontinuation. Neurologic and/or psychiatric side effects (11.1%, n=24) were the second most common type of patient-reported adverse effect in the subpopulation. These effects included a wide range of symptoms including mood disturbances (anxiety, depression, and suicidal ideation), headache, decreased libido, and numbness or tingling of the arm. Neurologic/psychiatric side effects were significantly associated with early discontinuation (P=0.02).

**Table 3:** Patient-reported adverse side effects by etonogestrel continuation rates Data were analyzed using chi-square, or Fisher exact tests as indicated.

Reported side effects	Continuation for ≤1 year (n=108)	Continuation for >1 year (n=108)	P value
All patient-reported adverse effects, n (%)	100 (92.6)	77 (71.3)	<0.001
Abnormal uterine bleeding	57 (52.8)	58 (53.7)	0.89
Genitourinary effects	4 (3.7)	0 (0)	0.12
Gastrointestinal effects	1 (0.9)	0 (0)	1.0
Neurologic/psychiatric effects	18 (16.7)	6 (5.6)	0.02
Dermatologic effects	4 (3.7)	3 (2.8)	1.0
Weight gain	8 (7.4)	8 (7.4)	1.0
Other	8 (7.4)	2 (1.9)	0.10

## Discussion

In this retrospective cohort study, we found that the one-year continuation rate of the etonogestrel implant in our population was significantly lower than the landmark CHOICE study rate of 83%, suggesting that continuation rates may vary widely among populations. Clinicians should be aware of this variance when prescribing LARC in their specific practice. Furthermore, early discontinuation in our study was associated with patient-reported adverse effects. A total of 92.6% of patients in the subanalysis group discontinued use at one year or less due to side effects.

Our findings may differ from the findings in the CHOICE study as individuals in the CHOICE study were enrolled prospectively, counseled uniformly about contraceptive choices, and contacted at pre-determined frequency intervals [8]. Our study population, in a real-world setting, was less likely to have been consistently monitored and may not have been uniformly counseled about contraception options and side effects.

While AUB was the most reported adverse effect in our study, AUB was not the most common reason mentioned for early discontinuation, suggesting that patients may be willing to tolerate some adverse effects in exchange for effective contraception. This may explain the increased continuation rates of the copper IUD compared to the implant reported by Moray et al. [5], for example, as the copper IUD is known to cause heavy and irregular bleeding and can even double the amount of menstrual blood loss a patient experiences compared with pre-insertion levels [11,12]. Thus, although previous literature has rightfully focused on the most common adverse effects of the implant, our study suggests future research should focus on the adverse effects most intolerable to the patient and likely to cause discontinuation. Perhaps future focus on the less common, but more troublesome, neurologic/psychiatric side effects is warranted. These effects can include headaches, a decrease in libido, and negative mood changes, all of which can have a significant impact on a patient's quality of life [13].

Study limitations include that some patients who ultimately removed their etonogestrel implant were not further evaluated for their reasoning behind the removal. Due to the retrospective nature of our study, counseling by providers on how to manage side effects was not available for our study. Also, our data were extracted from a limited population at one academic community hospital network, and therefore our results may not be widely generalizable to all patients who use the etonogestrel implant. However, any insight into potential avenues for increased LARC usage is valuable, as all this information may aid in achieving the ultimate goal of preventing unintended pregnancies and their serious consequences.

Future studies should aim to establish a baseline prevalence of mood disorders, headaches, and sexual dysfunction among patients eligible for LARC implants. By doing so, researchers may ascertain whether these are truly side effects of the implant or exacerbations of underlying conditions related or unrelated to implant placement. Identifying patients at an increased risk of neurologic and psychiatric side effects (i.e., those at an increased risk of early discontinuation) also may aid in appropriate pre-implant counseling and active management of pre-existing conditions, or, alternatively, counseling patients to select a different LARC that is more appropriate for their situation. Unintended pregnancy has far-reaching public health implications, from lower educational attainment and career advancement for those carrying the pregnancy to cycles of poverty and abuse. As of 2011, some 45% of all pregnancies in the US were unintended, with the highest rates among patients who had not completed high school, who identified as Black race, and who were of low socioeconomic status (<100% of the federal poverty line) [14]. These statistics reflect existing racial and wealth disparities in the US, underscoring the importance of access to effective contraception as a foundation of reproductive justice.

The WHO has asserted that a majority of unintended pregnancies could have been prevented with LARCs and that the status of women and the health of countries across the world may be dependent on access and utilization of devices like the etonogestrel implant [15]. Our study findings suggest adverse effects are powerful barriers to LARC usage, and providers should be intentional in adequately and carefully counseling patients on potential side effects before placement of the implant.

## Conclusions

The one-year continuation rate of the etonogestrel implant in our population is significantly lower than the value reported by CHOICE. Implant side effects are common and significantly affect rates of discontinuation. Our data suggest there is an opportunity for education and counseling for individuals opting for this LARC method. Providers must also understand the potential side effects of etonogestrel discontinuation in order to better counsel and select candidates for this type of contraception.
